# Draft Genome Sequence of the Marine Bacterium *Oceanimonas baumannii* ATCC 700832^T^

**DOI:** 10.1128/genomeA.01007-17

**Published:** 2017-09-07

**Authors:** William D. McClelland, Ariel M. Trachtenberg, Marc A. Brennan, Kyle S. MacLea

**Affiliations:** a Biology Program, University of New Hampshire, Manchester, New Hampshire, USA; b Biotechnology Program, University of New Hampshire, Manchester, New Hampshire, USA; c Department of Life Sciences, University of New Hampshire, Manchester, New Hampshire, USA

## Abstract

The aerobic phenol-degrading Gram-negative rod *Oceanimonas baumannii* ATCC 700832^T^ was first isolated from estuary mud from the River Wear, United Kingdom, in 1983. Information on the draft genome sequence for *O. baumannii* ATCC 700832^T^ is included in this announcement. The predicted genome size is 3,809,332 bp, with 55.88% G+C content.

## GENOME ANNOUNCEMENT

*Oceanimonas baumannii* strain GB6^T^ (=ATCC 700832^T^) is an aerobic Gram-negative rod that displays up to four polar flagella and can readily grow on minimal salts medium supplemented with phenol (1). It was isolated in 1998 from estuary mud at the mouth of the River Wear in Sunderland, United Kingdom (1). At the time of its discovery, Brown et al. found that *O. baumannii* should be classified in a new genus called *Oceanomonas* (1), later corrected in spelling to *Oceanimonas* (2), along with the type strain for this new genus, (*Pseudomonas*) *doudoroffii* ATCC 27123, now called *Oceanimonas doudoroffii* (1).

*O. baumannii* is a chemoorganotrophic gammaproteobacterium and has a requirement for sodium. It can grow at NaCl levels of up to 7% (wt/vol) (1) or 12% (3). Its absolute requirement for NaCl is 0.15% (wt/vol) (3), and it grows at temperatures between 10°C and 30°C (1). *O. baumannii* can be grown on glycerol, d-galactose, caprate, malate, citrate, ethanol, betaine, sarcosine, phenol, succinate, l-alanine, l-proline, and l-glutamate (1).

*O. baumannii* ATCC 700832^T^ was purchased from the ATCC (Manassas, VA, USA) in lyophilized form. It was rehydrated and then cultured in marine broth or agar (ATCC medium 2216) under conditions of 26°C for 48 h at atmospheric pressure. A single colony was picked and cultured in log-phase growth to obtain genomic DNA (gDNA), using the Genomic-tip 500/G kit (Qiagen, Valencia, CA, USA). gDNA was fragmented, tagged with adapters using the Nextera DNA library prep kit (Illumina, San Diego, CA, USA), and sequenced on an Illumina HiSeq 2500. Two hundred fifty-base-pair paired-end reads were generated at the Hubbard Center for Genome Studies (Durham, NH, USA), and Trimmomatic (4) was used for bioinformatic trimming and removal of adapter sequences prior to gene analysis.

The *O. baumannii* genome was assembled using SPAdes version 3.8.0 (5) from 2,790,420 reads, with an average length of 899 bp, into 70 contigs (after removal of small low-quality contigs). The total genome length was 3,809,332 bp, and we calculated an average coverage of 80×. The largest contig found was 408,079 bp, with a G+C content of 55.88% and an *N*_50_ value of 232,731 bp. The G+C content is in close agreement with that published by Brown et al., at 54% (1). The genome was annotated with the NCBI Prokaryotic Genome Annotation Pipeline (PGAP) process (6). PGAP found a total of 3,651 genes, 3,498 coding sequences (CDSs), 98 RNA genes, 55 pseudogenes, and 1 clustered regularly interspaced short palindromic repeat (CRISPR) array.

### Accession number(s).

This whole-genome shotgun project has been deposited at DDBJ/ENA/GenBank under the accession no. NQJF00000000. The version described in this paper is version NQJF01000000.
